# Fast PCA for processing calcium-imaging data from the brain of Drosophila melanogaster

**DOI:** 10.1186/1472-6947-12-S1-S2

**Published:** 2012-04-30

**Authors:** Martin Strauch, C Giovanni Galizia

**Affiliations:** 1 Bioinformatics and Information Mining, University of Konstanz, 78457 Konstanz, Germany; 2 Neurobiology, University of Konstanz, 78457 Konstanz, Germany

## Abstract

**Background:**

The calcium-imaging technique allows us to record movies of brain activity in the antennal lobe of the fruitfly *Drosophila melanogaster*, a brain compartment dedicated to information about odors. Signal processing, e.g. with source separation techniques, can be slow on the large movie datasets.

**Method:**

We have developed an approximate Principal Component Analysis (PCA) for fast dimensionality reduction. The method samples relevant pixels from the movies, such that PCA can be performed on a smaller matrix. Utilising *a priori *knowledge about the nature of the data, we minimise the risk of missing important pixels.

**Results:**

Our method allows for fast approximate computation of PCA with adaptive resolution and running time. Utilising *a priori *knowledge about the data enables us to concentrate more biological signals in a small pixel sample than a general sampling method based on vector norms.

**Conclusions:**

Fast dimensionality reduction with approximate PCA removes a computational bottleneck and leads to running time improvements for subsequent algorithms. Once in PCA space, we can efficiently perform source separation, e.g to detect biological signals in the movies or to remove artifacts.

## Introduction

The fruitfly *Drosophila melanogaster *is a model organism for research on olfaction, the sense of smell. Calcium-imaging, i.e. microscopy with fluorescent calcium-sensitive dyes as reporters of brain activity, allows us to answer questions on how information about odors is processed in the fruitfly's brain [1].

**Figure 1:**
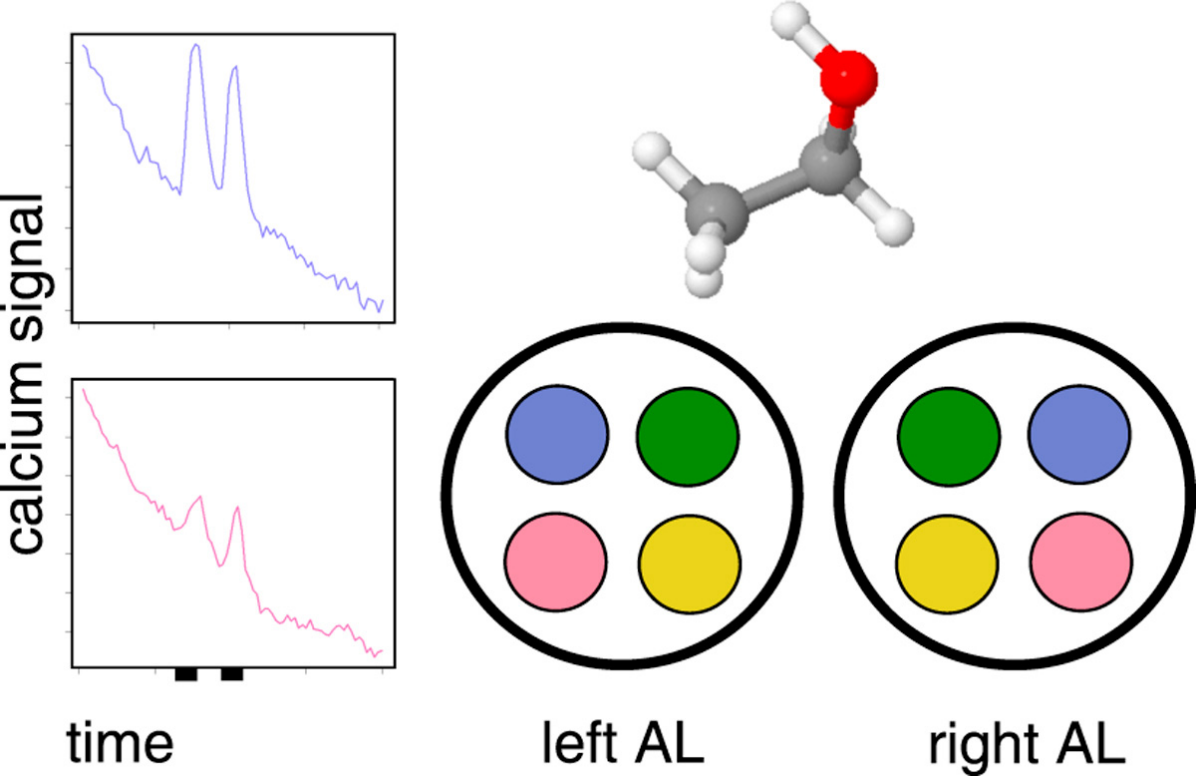
**Odor coding**. An odor molecule is encoded as a pattern of glomerulus responses in the ALs of the fruitfly brain. The green and yellow glomeruli remain inactive (not shown), whereas the blue and magenta glomeruli respond to the odor presentations (black bars mark two pulses of 1s each) with differential strength. Left and right ALs, that receive input from the left and right antennae, are mirror-symmetric and contain the same types of glomeruli.

The datasets we consider are *in vivo *calcium-imaging movies recorded from the antennal lobe (AL). Here, information from the odor receptors on the antennae is integrated, processed and then relayed to higher-order brain regions. In the AL, each odor smelled by the fly is represented as a spatio-temporal pattern of brain activity (see schematic in Figure 1). The coding units of the AL are the so-called glomeruli that exhibit differential responses to odorants. The combined response of all the ca. 50 glomeruli in a single fruitfly AL forms an odor-specific pattern [2].

A major objective of biological research in this field is to map the *Drosophila *olfactome, i.e. odor representation and similarity as sensed by *Drosophila*. Odor response patterns recorded so far are available in the DoOR database [3].

In terms of data analysis, our goal is to extract glomerular signals and patterns from calcium-imaging movies. Ideally, we would like to do this in a fast and memory-efficient way, keeping in mind that the size of the movies is going to increase further in the future due to the advent of high-resolution and three-dimensional 2Photon microscopy [4].

Here, we process imaging movies from the *Drosophila *AL with Independent Component Analysis (ICA) [5]. Source separation with ICA has proven helpful in the analysis of brain imaging data [6-8], and can be employed to "find" glomeruli in calcium-imaging movies, i.e. to separate their signals from noise and artifacts [7].

ICA algorithms are typically performed after decorrelation and dimensionality reduction with a Principal Component Analysis (PCA) [9,10], delegating the main computational load to the PCA pre-processing step [6,7,11,12]. While PCA is generally feasible from a computational point of view, the standard approach to PCA by Singular Value Decomposition (SVD) [13] of the data matrix scales quadratically with the number of columns (or rows), and can be slow on the large movies files.

We thus propose an approximate solution to PCA that, while being substantially faster than exact PCA, keeps biological detail intact. Apart from our specific ICA application, fast dimensionality reduction is also of general utility for computations on imaging movies.

How do we achieve a high-quality approximation to PCA? The observation is that, after processing, we usually deem only a small fraction of the pixels to be relevant, while many others do not report a biological signal. Following a feature selection paradigm [14], we could, at some computational expense, optimise a small set of most relevant pixels as input for PCA.

Instead, we propose to quickly select not few but many pixels (out of many more), and we do so by investing a small amount of time into computing pixel sampling probabilities that allow us to pick relevant pixels preferentially. Evaluation of a pixel's relevance relies on *a priori *knowledge about the nature of the biological sources: signals from neighbouring pixels in the regions of interest, the glomeruli, are correlated.

We proceed as follows: In the methods section, we first introduce our notation and summarise prior work. We then consider a general framework for approximate SVD and modify it for our approximate PCA that is explicitly designed for the imaging movies. In the results section, we provide a technical evaluation with respect to speed and accuracy of the results, as well as practical examples for the fast analysis of *Drosophila *imaging data with approximate PCA followed by ICA.

## Methods

### Preliminaries

#### Notation

PCA [9,10] provides the following low-rank approximation to a data matrix *A *based on orthogonal basis vectors, the "lines of closest fit to systems of points in space" [9], so-called principal components:

(1) $$A^{m\times n}:A_{k}=T^{m\times k}\;S^{k\times n}=\overset{k}{\underset{r=1}{\sum }}T_{Ir}\;S_{rJ}$$

For our purposes, *A *is the calcium-imaging movie with *m *timepoints and *n *pixels (images flattened into vectors). Consequently, the rank-*k *approximation *A_k _*consists of a matrix *T *with a temporal interpretation (distribution of loadings, timeseries) and a matrix *S *with a spatial interpretation (principal component images). Regarding notation, we refer to the *j*th column of *A *as *A_Ij_*, and denote the element at the intersection of the *i*th row and the *j*th column as *A*_*i, j*_. When we refer to column selection from matrix *A*, we select pixels, or, more precisely, pixel-timeseries vectors of length *m*.

#### Computing PCA and features for PCA

PCA can be computed by a singular value decomposition (SVD): *A *= *U*Σ*V *[13]. SVD is a minimiser of ||*A *- *A_k_*||*_Fr_*, i.e. the error incurred by a rank-*k *approximation *A_k _*to matrix *A *with respect to the Frobenius norm. When the data is centered, which we can assume as our algorithms require one pass over the matrix prior to PCA, the top-*k *right singular vectors *V *correspond to the top-*k *principal components [15]. The usual approach is to compute the SVD with full dimensionality in *V *, which is then truncated to the top-*k *singular vectors with highest singular values. In contrast, NIPALS-style PCA [16,17] (s.a. Algorithm 3) computes only the top-*k *components. Another approach to PCA is the eigenvalue decomposition of the covariance matrix [10].

Regarding feature selection for PCA, Jolliffe [18,19] provided evidence that many variables can be discarded without significantly affecting the results of PCA. Several methods based on clustering or multiple correlation were tested in these studies aimed at selecting few non-redundant features in a PCA context. Similar, more recent work was performed by Mao [20] and Li [21].

A paper on feature selection for PCA by Boutsidis et al. [14] guarantees an error bound for the approximate solution to PCA based on a subset of the columns of matrix *A*. While conceptually related to the randomised framework discussed below, running time is in fact slightly above that of PCA, the objective being not speedup but identifying representative columns for data analysis.

#### Source separation with ICA

On imaging movies, source separation with ICA can be cast into the same notation as PCA (1). Where PCA relies on orthogonal, i.e. uncorrelated basis vectors, the goal of ICA [5] is to find statistically independent basis vectors, i.e. independent timeseries in *T*, or independent images in *S*. ICA falls into the category of "blind source separation" (BSS). It tries to unmix signal sources, such as glomerular signals, artifacts and noise, mostly blind with respect to the nature of both signals and mixing process, based solely on a statistical model. The model assumption behind ICA is that the sources are (approximately) independent and (for all but one source) non-Gaussian.

ICA can detect the glomerular sources in calcium-imaging movies [7] and therefore serves as an application example: it is useful to compute ICA on such movies and we can solve the unmixing problem much more efficiently if we first perform fast dimensionality reduction with approximate PCA. We employ one of the most common ICA algorithms, the fixed-point iteration *fastICA *[5,22].

### Monte Carlo approximate SVD

Here, we rely on a Monte Carlo-type approximate SVD proposed by Drineas et al. [23,24]. Randomly selecting *c *columns from *A *into *C*^*m*×*c*^, we can achieve an approximation to the sample covariance of *A *with an error of ||*AA^T ^*- *CC^T^*||*_Fr_*.

In [24], the following relationship between the optimal rank-*k *matrix *A_k_: *= SVD(*A*) and the approximation *H_k_: *= SVD(*C*) was shown:

(2) $$\begin{array}{c} \;\;\;\;∥A-H_{k}H_{k}^{T}A{∥}_{Fr}^{2}\;\leq \\ ∥A-A_{k}{∥}_{Fr}^{2}+2\sqrt{k}∥AA^{T}-CC^{T}{∥}_{Fr} \end{array}$$

The error of the approximate SVD of *A *thus depends on the optimal rank-*k *approximation *A_k _*from exact SVD plus the difference in covariance structure due to column sampling. The factor $2\sqrt{k}$ reveals that the error bound is tighter for small *k*, implicating that, if larger *k *are desired, we should attempt to reduce the error ||*AA^T ^*- *CC^T^*||*_Fr _*, e.g. by selecting more columns.

The main result of [24] was that, given appropriate sampling of *c *columns from *A*, the expected error with respect to the Frobenius norm of *A *is *ε*:

(3) $$E\left[∥A-H_{k}H_{k}^{T}A{∥}_{Fr}^{2}\right]\;\leq \;∥A-A_{k}{∥}_{Fr}^{2}+\;\varepsilon ∥A{∥}_{Fr}^{2}$$

This result holds for column sampling probabilities *p_j _*that are not uniform, but depend on the euclidean column norms *|A_Ij_|*:

(4) $$p_{j}^{norm}=\frac{{|A_{Ij}|}^{2}}{∥A{∥}_{Fr}^{2}}$$

In particular, the upper bound from (3) holds if we sample with replacement $c\geq \frac{4k}{\varepsilon^{2}}$ columns. This means that the error *ε *can be made arbitrarily small by sampling a sufficient number of columns *c*, and we can compute in advance the *c *required to achieve the desired *ε*.

Following the Monte Carlo framework, we can sample *c *pixel-timeseries into *C *and achieve an upper bound on the error by approximate SVD with respect to $∥A{∥}_{Fr}^{2}$ and the approximation of the time *× *time covariance *AA^T^*.

The upper bound, is, however, not very tight. If we wish to achieve *ε *= 0.05 for *k *= 20, we would need to sample with replacement 32, 000 pixels, which leads to considerable speedups on large datasets (≈ 150, 000 pixels), but is impractical for the medium-size datasets (≈ 20, 000 pixels).

The main contribution of the norm-based Monte Carlo approach is thus to show that the correctness of SVD/PCA does not collapse under pixel sampling, but that the error is rather asymptotical and can be decreased further and further by sampling more pixels.

### Covariation sampling

Although this pixel sampling may work well in practice, the theoretical bound is not very tight. Can we then more explicitly select biologically relevant pixels so as to ensure our confidence in the fast approximation?

The intuition is, that, if our pixel sample covers all glomeruli, the "biological error" will be small. We thus motivate a biological criterion, covariation between neighbouring pixel-timeseries, as an importance measure. The assumption we rely on is about the spatial aspect of the data, namely that a glomerulus in an imaging movie covers several adjacent pixels that all report the same signal (plus noise). This *a priori *knowledge is also exploited in the "manual" analysis of imaging movies by visualising the amount of neighbourhood correlation for each pixel (see for example Figure 2 in [25]).

**Figure 2:**
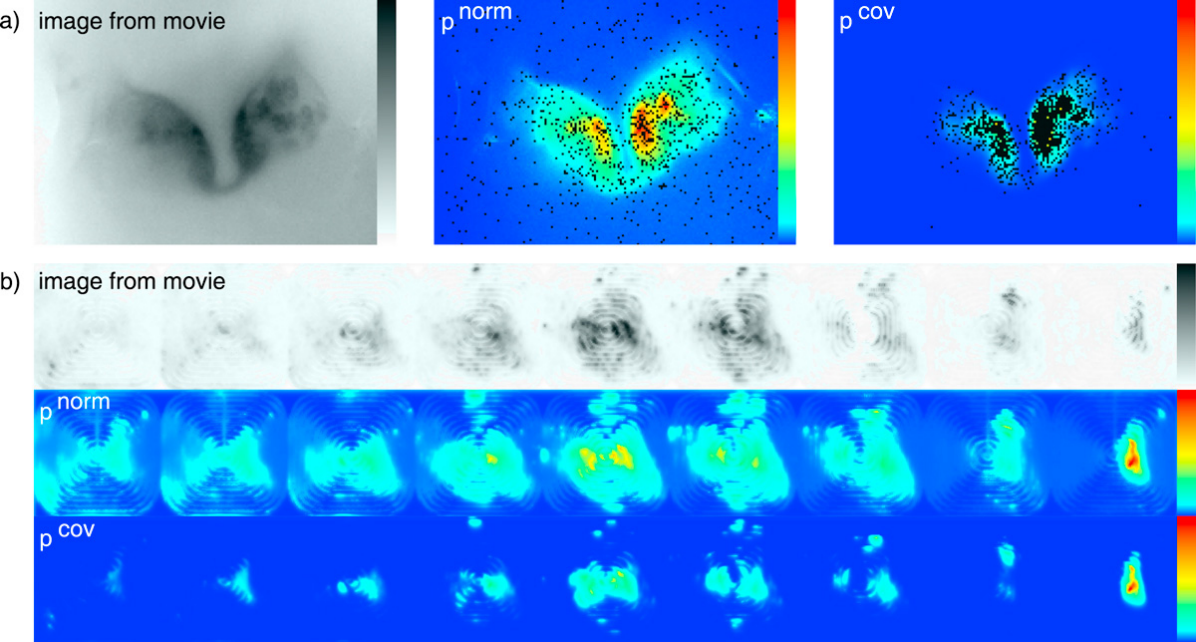
**Probability distributions**. **a) **Image from the Drosophila2D movie, distribution of norm probabilities and distribution of covariation probabilities. A 5% pixel sample (Algorithm 1 for norms, Algorithm 2 for covariance) is superimposed in black. **b) **Drosophila3D. For visualisation, we discretised the continuous z-axis into 9 layers.

Our approach is to compute a small part of the pixels *× *pixels covariance matrix exactly, and then to sample those pixels that contribute much to the norm of this matrix. We are interested in the local part of the sample covariance matrix which we denote as *L *= *f *(*A^T ^A*), *f *(*X*_*i, j*_) being defined as follows:

(5) $$f\left(X_{i,j}\right)=X_{i,j}\;\mathrm{if}\text{ pixels i and j are neighbours, }\mathrm{else}\text{ 0}$$

The column norms of *L*^*n*×*n *^correspond to the amount of covariation with neighbouring pixels, i.e. if the column is from within one of the spatially local sources (glomeruli), the norm is high. Consequently, if we apply the column norm sampling according to (4) not to the movie matrix *A *but to the derived matrix *L*, we will more explicitly select columns with biological signal content.

Departing from the error bound scheme regarding the norm, we can now estimate in advance the biological signal content by computing for how much of ||*L*||*_Fr _*the pixel sample accounts. In the results section we will see that small pixel samples can explain a large part of ||*L*||*_Fr_*.

In practice, it is more convenient not to construct the entire matrix *L*, but to directly compute the column norms of *L *on the movie *A*. Here, the index *r *enumerates the 8 immediate neighbour pixels of the pixel in column *j*, i.e. the pixels (*x*, *y - *1), (*x*, *y + *1), etc. in x/y coordinates of the (unflattened) images.

(6) $$|L_{Ij}|\;=\sqrt{\underset{r}{\sum }{\left(A_{Ij}\;A_{Ir}\right)}^{2}}$$

Sampling from *L *with norm probabilities (4) amounts to sampling from *A *with covariation probabilities *p^cov^*, where $∥L{∥}_{Fr}=\sqrt{{\sum_{j}\sum_{r}|A_{Ij}\;A_{Ir}|}^{2}}$ can be computed on the fly while computing the column norms.

(7) $$p_{j}^{cov}=\frac{|L_{Ij}{|}^{2}}{∥L{∥}_{Fr}^{2}}$$

### Fast PCA for calcium-imaging movies

We first propose two alternative methods for pixel sampling (Algorithm 1 and 2) which we then utilise to perform PCA on a small matrix (Algorithm 3). Sampling allows for an adaptive resolution without a sharp cutoff by a threshold.

#### Pixel sampling

In Algorithm 1, we sample exactly *c *pixel-timeseries with replacement from the movie matrix *A *and scale them as in the Monte Carlo framework [24]. We employ norm-based probabilities (4), such that we can make use of the theoretical upper bounds.

**Algorithm 1 Pixel sampling with replacement**, *input*: movie matrix *A ∈ *ℝ^*m*×*n*^, number of pixels *c*, norm probabilities *p^norm ^*= (*p*_0_,..., *p*_(*n *- 1)_), *output*: sample matrix *C *∈ ℝ^*m*×*c*^

**for all ***t *∈ [1, *c*] **do**

pick column *j *from *A *with probability *p_j_*

$C\;\left[\;,\;t\right]:=A\;\left[\;,\;j\right]\;1/\sqrt{cp_{j}}$

end for

The above sampling strategy is necessary for the Monte Carlo scheme to work, however, for the covariation probabilities (7), the most parsimonious approach is simply sampling without replacement: Algorithm 2.

**Algorithm 2 Pixel sampling without replacement**, *input*: movie matrix *A *∈ ℝ^*m*×*n*^, number of pixels *c*, covariation probabilities *p^cov ^*= (*p*_0_,..., *p*_(*n *- 1)_), *output*: sample matrix *C *∈ ℝ^*m*×*c*^

R: = {}

**for all ***t *∈ [1, *c*] **do**

sample *j *∉ *R *from *A *with probability *p_j_*

*C*[, *t*]: = *A*[, *j*]; R: = R ∪ j;

end for

Note that we can generally assume absence of movement, i.e. pixel identity remains the same throughout the measurement. The AL is a fixed anatomical structure, and small-scale movement that leads to shaky recordings can be eliminated by standard image stabilisation (as e.g. in [1]).

#### Computing PCA

We employ NIPALS-style PCA [16,17] for computing the top-*k *components. Complexity for NIPALS-style PCA is O(mnki) for *k *principal components and *i *iterations until convergence of the components. Typically, *k *and *i *are small numbers (*i *≈ 5 - 10). In contrast, SVD with a space and time complexity of $O\left(\text{min}\left(n^{2}m,\;nm^{2}\right)\right)$ is generally not efficient. In particular, the number of timepoints *m *can still be the smaller dimension after sampling.

Note that Drineas et al. [24] assume that SVD is used for *H_k_: *= SVD(*C*), however proofs for the error bounds do not depend on algorithm structure but rather on the eigenvalue spectrum.

We have summarised the approach in Algorithm 3. The first step consists of running Algorithm 1 or 2 in order to obtain the *n *× *c *sample matrix *C*. To achieve the PCA decomposition (1), we then sequentially compute the top-*k *components in *T *and obtain full-size images in *S *by *S: *= *T*^+ ^*A*, where *T*^+ ^is the generalised Moore-Penrose pseudoinverse of *T*.

The approximate PCA requires O(mcki) only for the timeseries in *T *and O(mcki+mnk) for both timeseries and images. On top of that, we need O(n) for precomputing the probabilities. In practice, we also profit from the redistribution of the computational load, which allows for greater speedups: unlike sequential PCA computation, the final matrix multiplication is highly parallelisable.

**Algorithm 3 Approximate PCA**, *input: A *∈ ℝ^*m*×*n*^, number of samples *c*, number of components *k*, *output: T *∈ ℝ^*m*×*k *^, *S *∈ ℝ^*k*×*n*^

select *c *columns from *A *into *C *with Algorithm1 or Algorithm2

//compute NIPALS-style PCA on matrix *C*

**for all ***l *∈ [1, *k*] **do**

$t_{l}:=argmax_{\left(C_{Ij}\in R\right)}∥C_{Ij}∥$

**while **not converged **do**

$s_{l}:=C^{T}t_{l}/\text{(}t_{l}^{T}t_{l}\text{);}\;\;t_{l}:=\left(Cs_{l}\right)/\left(s_{l}^{T}s_{l}\right)$;

end while

$C:=C-t_{l}^{T}s_{l};\;\;T\left[\;,\;l\right]:=t_{l}$;

end for

//compute full-size images

*S: *= *T*^+ ^*A*

## Results

### Datasets and pixel selection strategies

Our test datasets are "Drosophila2D" (Figure 2a: left and right *Drosophila *AL; light microscopy, staining with G-CaMP dye, 19, 200 pixels × 1, 440 timepoints), and "Drosophila3D" (Figure 2b: single *Drosophila *AL; three-dimensional 2Photon microscopy, G-CaMP, 147, 456 pixels × 608 timepoints).

Both datasets are concatenations of multiple measurements. In the middle of each measurement (except for controls), an odor was presented to the fly. A series of different odors was employed which enables us to tell apart glomeruli based on their differential response properties.

In Figure 2, we give also visual examples for the probability distributions. In contrast to the norms, covariance probabilities are concentrated on few regions, which can be sampled very densely even with small *c*.

### Empirical evaluation

As evaluation criteria we rely on the Frobenius norm error ||*A *- *TS*||*_Fr _*= ||*A *- *A_k_*||*_Fr _*as a standard measure for low-rank approximation, and on the biologically motivated *covariation energy*, the amount of local covariation accounted for by the pixel sample (unique column indices in *R*):

(8) $$\left(\overset{R_{c}}{\underset{t=R_{1}}{\sum }}{|L_{It}|}^{2}\right)/∥L{∥}_{Fr}^{2}$$

**Figure 3:**
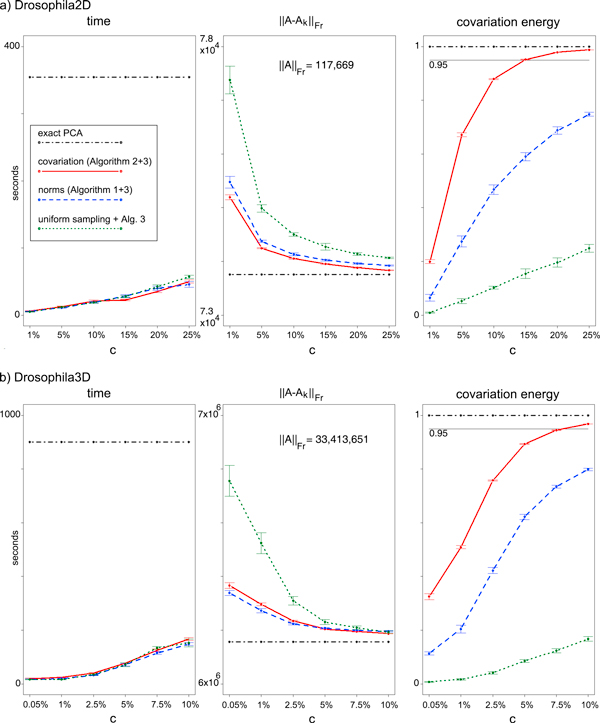
**Performance**. Means and standard deviations for time and error measures (10 repetitions) for exact and approximate PCA. Number of pixels *c *is given in % of the total number *n*. Running times (Intel Core Duo T6400, 2GHz) are for the entire Algorithm 3, including computation of probabilities. All measurements are for rank-*k *= 30 approximations, as we found that 20-30 components are typically sufficient to detect all glomeruli. Lower principal components only explain more of the noise (see also Figure 4).

Results are presented in Figure 3. As baselines, we give results from exact NIPALS-style PCA and approximate PCA with uniform pixel sampling. All algorithms were implemented in Java, using the Parallel Colt library [26].

Already small samples lead to low additional error with respect to the Frobenius norm. E.g., on the Drosophila2D dataset, exact PCA achieves a Frobenius norm error of 73, 754.64 for a rank-*k *= 30 approximation, where ||*A*||*_Fr _*= 117, 668.99. In comparison, covariation sampling with Algorithm 2 achieves a Frobenius norm error of 75, 187.93 based on only 1% of the pixels.

Both, norm error and covariation energy, reach about the level of accuracy of exact PCA already with sample sizes of between 10% to 15% of the pixels, whereas time consumption grows only slowly (Figure 3). Generally, sampling based on norms or covariation is superior to uniform pixel sampling, and the covariation sampling with Algorithm 2 accumulates more covariation energy in smaller samples than the other strategies. Error bars for Algorithm 1 and 2 are small, indicating that results are reproducible despite of the randomised techniques.

How many pixels do we need to sample? While our empirical measurements suggest that between 10% to 15% of the pixels are sufficient, even smaller samples of about 1% of the pixels give good results in practice, the error being already much lower than the expected upper bounds. As a "safe" strategy we suggest to sample pixels with Algorithm 2 until the cumulated covariation energy exceeds a threshold, e.g. 0.95 (straight line in Figure 3).

**Figure 4:**
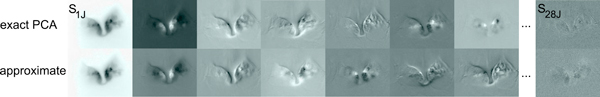
**Example for PCA**. Top principal components computed by exact PCA and approximate PCA with covariation probabilities (1% pixel sample).

To give a visual impression of how the technical quality measures translate into image quality, we compare principal component images in *S *that were computed with exact and approximate PCA (Figure 4). Both span approximately the same space, however, due to the different input matrices, there is not necessarily a one-to-one correspondence.

### Application example: ICA

Recall that both PCA and ICA result in a decomposition of the form *A_k _*= *T ^PCA ^S^PCA^*, or *A_k _*= *T ^ICA ^S^ICA^*, respectively. As input for ICA, we can either take the principal component images in *S^PCA ^*or the principal component timeseries in matrix *T^PCA^*.

**Figure 5:**
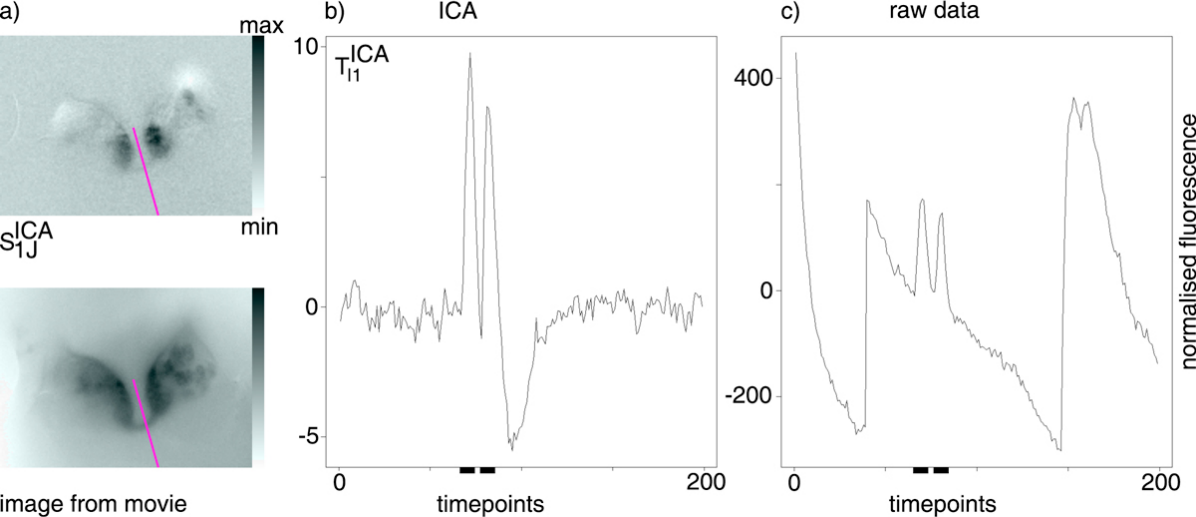
**Example for temporal ICA**. Performing ICA on the principal component timeseries matrix *T^PCA^*. **a) above: **spatial component $S_{1J}^{ICA}$ that contains a glomerulus pair (black pixels); **below: **image from raw movie, indicating the shapes of the left and right ALs. **b) **Timeseries component $T_{I1}^{ICA}$ (that corresponds to $S_{1J}^{ICA}$) on a 200-timepoints interval including a double odor presentation (marked by the bars). **c) **For comparison, we show the mean timeseries for the glomerulus pair on the raw movie *A*.

In Figure 5a we give an example for temporal ICA on principal component timeseries (Drosophila2D data, covariation probabilities, *c *= 0.15*n*). Here, the highest (black) coefficients in the image $S_{1J}^{ICA}$ indicate the positions of a glomerulus pair, the same type of glomerulus in the left and right AL. Both AL halves are mirror-symmetric and each contain a full set of glomeruli. Judging from their positions, the two glomeruli are very likely a pair, i.e. both receive input from the same types of receptor neurons and therefore have equal (plus noise) response properties.

Taking into account the corresponding timeseries in $T_{I1}^{ICA}$ (Figure 5b), we can assume that we indeed have found glomeruli and not some other pair of objects: we see a double response to the double odor stimulation, where a response is a sharp increase in fluorescence, followed by a decline below baseline.

For comparison, we extracted (by thresholding) positions of all black pixels in $S_{1J}^{ICA}$ and computed their mean timeseries on the raw movie *A*, i.e. the raw signal of the glomerulus pair: Figure 5c. Here, we can see that the movie consists of a concatenation of measurements that each exhibit a strong trend: the dye bleaches due to measurement light, an artifact which is absent in the ICA component.

**Figure 6:**
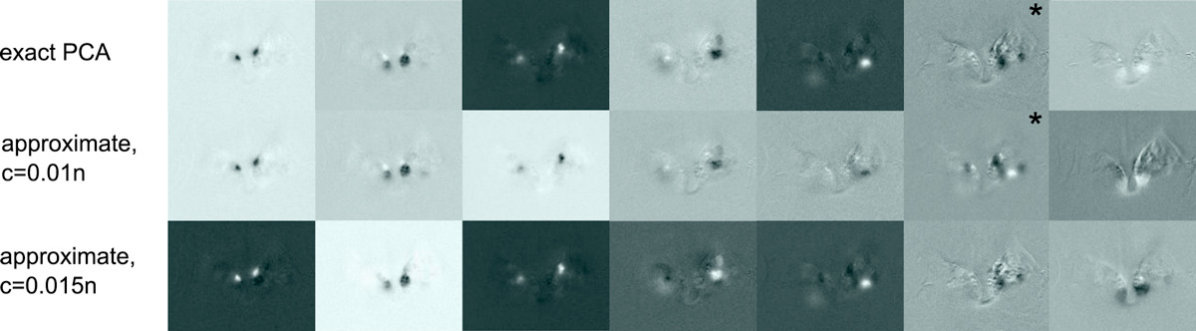
**Example for spatial ICA**. Performing ICA on the principal component images matrix *S^PCA^*. We show all spatial independent components that capture glomeruli. **Top: **ICA was run after exact PCA, **bottom: **ICA was run after approximate PCA with a 1% or 15%, respectively, pixel sample (covariation probabilities). Closest matches are placed in the same column.

As another example, we have applied spatial ICA, working on *S^PCA ^*as input. This can be helpful to find glomerulus positions in order to construct a glomerulus map [7]. In Figure 6, we show all independent component images from *S^ICA ^*that "contain" glomeruli. Note that the sign is arbitrary in an ICA decomposition [5], i.e. glomeruli can appear black on white or vice versa. Based on approximate PCA we can detect all but one (marked with a star) component already with a 1% pixel sample, whereas with a 15% sample we can also recover the missing component.

Here, we have regarded the spatial and temporal aspect of the data separately leading e.g. to spatial components that are not entirely local (Figure 5a). For future applications, it might be helpful to consider a spatio-temporal criterion [11,12] that balances between spatial and temporal independence of the sources.

## Conclusions

We have shown that source separation can, in principle, detect glomerulus positions and remove artifacts in *Drosophila *imaging movies. Many source separation algorithms exist that optimise different criteria and it remains subject to further research which method is most robust for a particular data type.

Here, we have concentrated on finding a fast approximate solution to PCA that reduces data size prior to source separation. Delegating the main computational load to the preprocessing with fast PCA allows any source separation algorithm to scale up easily with the growing data sizes in imaging. A further promising area of application is, with due modifications, online analysis such that denoised movies are available already during the course of the experiment.

Our strategy for fast approximate PCA relies on simple precomputations that can be performed in a single pass over the data. Based on *a priori *knowledge and the information gathered in this step, we can sample pixels from the movie in order to perform exact PCA much more efficiently on a smaller matrix. Sampling with norm probabilities gives rise to an upper bound for the expected error. Sampling with covariation probabilities, we can ensure a high-quality approximation by requiring a high amount of covariation energy in the sample.

Our empirical results show that small pixel samples reliably lead to approximations with low error. It remains as an interesting question for further research, whether it is possible to translate these results into theory, e.g. by proving tight error bounds that incorporate the *a priori *knowledge.

## Competing interests

The authors declare that they have no competing interests.

## Authors' contributions

MS performed research and wrote the manuscript. CGG supervised research and edited the manuscript. All authors read and approved the final manuscript.
